# Rad51 Activates Polyomavirus JC Early Transcription

**DOI:** 10.1371/journal.pone.0110122

**Published:** 2014-10-13

**Authors:** Martyn K. White, Rafal Kaminski, Kamel Khalili, Hassen S. Wollebo

**Affiliations:** Center for Neurovirology, Department of Neuroscience, Temple University School of Medicine, Philadelphia, Pennsylvania, United States of America; University of Regensburg, Germany

## Abstract

The human neurotropic polyomavirus JC (JCV) causes the fatal CNS demyelinating disease progressive multifocal leukoencephalopathy (PML). JCV infection is very common and after primary infection, the virus is able to persist in an asymptomatic state. Rarely, and usually only under conditions of immune impairment, JCV re-emerges to actively replicate in the astrocytes and oligodendrocytes of the brain causing PML. The regulatory events involved in the reactivation of active viral replication in PML are not well understood but previous studies have implicated the transcription factor NF-κB acting at a well-characterized site in the JCV noncoding control region (NCCR). NF-κB in turn is regulated in a number of ways including activation by cytokines such as TNF-α, interactions with other transcription factors and epigenetic events involving protein acetylation – all of which can regulate the transcriptional activity of JCV. Active JCV infection is marked by the occurrence of rapid and extensive DNA damage in the host cell and the induction of the expression of cellular proteins involved in DNA repair including Rad51, a major component of the homologous recombination-directed double-strand break DNA repair machinery. Here we show that increased Rad51 expression activates the JCV early promoter. This activation is co-operative with the stimulation caused by NF-κB p65, abrogated by mutation of the NF-κB binding site or siRNA to NFκB p65 and enhanced by the histone deacetylase inhibitor sodium butyrate. These data indicate that the induction of Rad51 resulting from infection with JCV acts through NF-κB via its binding site to stimulate JCV early transcription. We suggest that this provides a novel positive feedback mechanism to enhance viral gene expression during the early stage of JCV infection.

## Introduction

The human neurotropic polyomavirus JC (JCV) causes the fatal demyelinating disease of the central nervous system (CNS) known as progressive multifocal leukoencephalopathy (PML) [1]. Primary infection by JCV is very common, usually occurs early in life and appears to be subclinical so that the only evidence for infection is the appearance of serum antibodies to the virus (reviewed in [2]). However, it is clear that the virus persists after infection since it may be shed episodically in the urine and the virus can reappear under conditions of severe immune impairment and productively infect the astrocytes and oligodendrocytes in the CNS giving rise to multiple regions of demyelination and causing PML. PML is almost always associated with some form of impaired immune function including HIV-1/AIDS [3], treatment with Natalizumab [4]–[7] Rituximab [8], Efalizumab [9] or immunosuppressive drugs administered to prevent transplant rejection [10], [11] as well as lymphoproliferative and myeloproliferative disorders [12] and other instances of chronic immunosuppression (reviewed in [13], [14]). Our understanding of the pathogenesis of PML and the molecular events of the JCV life cycle remains incomplete. For example, the molecular basis and site(s) within which latent/persistent virus exists and the mechanism whereby the virus reactivates to cause PML remain controversial (reviewed in [2], [15]).

JCV is a small DNA tumor virus belonging to the Polyomavirus family that has a circular, closed, supercoiled DNA genome and is small in size (∼5.1 Kbp). Both JCV and Polyomavirus BK (BKV), which causes BKV-associated nephropathy, were discovered in 1971 and for many years they were the only known human polyomaviruses, until about 6 years ago when a series of novel polyomaviruses were discovered and now there are at least ten [16]. The genome of JCV is comprised of two coding regions, early and late, which are transcribed in opposite directions [17], [18]. The coding regions are separated by the noncoding control region (NCCR), which functions as a bidirectional promoter and contains the binding sites for many transcription factors that regulate JCV gene expression as well as the origin of viral DNA replication. The NCCR co-ordinates the expression of the early proteins (large T-antigen and small t-antigen) and late proteins (VP1, VP2, VP3 and agnoprotein) during the stages of the viral life cycle. The binding of various cellular and viral transcription factors to the NCCR regulates these transcription programs [19].

Our earlier work implicated the NF-κB signaling pathway as a key regulator of the transcriptional status of JCV [20]–[25]. A unique binding site for NF-κB is located in the early proximal side of the JCV NCCR and is positively regulated by NF-κB p65 binding and negatively regulated by isoforms of the C/EBPβ protein, which bind to an adjacent site [22]. We have also found that TNF-α stimulated JCV transcription through this element [24] and that it is also a target of calcineurin/NFAT4 signaling [25]. The histone deactylation inhibitor trichostatin A (TSA) and expression of the transcriptional coactivators/acetyltransferase p300 were also found to activate transcription via the NF-κB binding site indicating that epigenetic events involving protein acetylation are also important [26]. Our recent data reported here indicate the involvement of Rad51 in this signaling axis.

Rad51 is a highly conserved protein that functions in the homologous recombination-directed DNA double-strand break repair pathway [27]. Infection of astrocyte cultures by JCV results in the induction of DNA and genome damage as evidenced by changes in ploidy, increased micronuclei formation and an induction of the levels of phospho-histone2AX (γH2AX), a marker for double-strand breaks. Concomitantly, JCV infection also causes an induction in the levels of some DNA repair enzymes, notably a large elevation in Rad51 [28]. In other experiments, we have also found that Rad51 is able to bind and activate NF-κB p65 in HIV-1-infected human microglial cells [29] and interplay of Rad51 with the NF-κB signaling pathway stimulates HIV-1 gene expression while inhibition of Rad51 function represses HIV-1 infection [30]. In the light of these findings, we investigated a role for Rad51 on the transcription and replication of JCV. Rad51 was found to act through NF-κB via its binding site in the JCV NCCR to activate JCV early transcription suggesting a positive feedback mechanism to enhance viral gene expression during the early stage of JCV infection. The function of Rad51 in cellular DNA repair and maintenance of cell homeostasis is well established and it is tightly regulated throughout the cell cycle. However in the context of JCV infection, Rad51 may be co-opted by the virus to facilitate the events that ultimately lead to cell lysis.

## Materials and Methods

### Cell culture and plasmids

The human TC620 oligodendroglioma cell line [24] and SVG-A, a human cell line which was derived from primary human fetal glial cells transformed by origin-defective SV40 and expresses SV40 T-Ag [31], were maintained in Dulbecco’s Modified Eagle’s Medium (DMEM) supplemented with 10% fetal bovine serum (FBS) line as we have previously described [24]. Reporter plasmids for the wild-type JCV early promoter (JCV_E_-LUC) and promoter mutants m1 and m2, which contained mutations at two adjacent sites within the KB site, have been described previously [22]. The expression plasmids pCMV-p65, pCMV-LIP [22] and pCMV-Rad51 [29] were described previously. Dominant negative IκBα expression plasmid (DN-IαKB) was from Clontech (pCMV-IκBαM, where IκBαM differs from IκBα by Ser-to-Ala mutations at residues 32 and 36). pJCV has the JCV Mad-1 wild-type whole genome DNA cloned into the *Bam*HI site of pBluescript KS.

### Antibodies

Rabbit polyclonal anti-p65 (c-20, sc-372, Santa Cruz Biotechnology Inc., Santa Cruz, CA) and mouse monoclonal anti-C/EBPβ (H7, sc-7962, Santa Cruz) which recognizes all three C/EBPβ isoforms were used for Western blots. Rabbit polyclonal anti-Rad51 antibody (D4B10) was from Cell Signaling Technology, Inc. except for immunocytochemistry where mouse monoclonal anti-Rad51 antibody was used (14B4, GeneTex Inc.) Mouse monoclonal anti-α-tubulin (clone B512) was from Sigma (St. Louis, MO). Rabbit monoclonal antibody (C5811) to acetyl-histone H3 (K9) was from Cell Signaling, Inc., Danvers MA. We have previously described mouse monoclonal antibody against JCV VP1 [32].

### Western blots

Western blots were as previously described [33] except antibody was detected with the LI-COR system. Blots were incubated with IRDye 800CW Goat Anti-Rabbit and IRDye 680RD Goat Anti-Mouse Li-COR dyes and visualized with an Odyssey CLx Imaging System (LI-COR, Inc., Lincoln, NE) using LI-COR Odyssey software. Band intensities were quantified using the Quantity One software (Bio-Rad, Hercules CA).

### Transient transfection and reporter assays

Experiments involving co-transfection of reporter plasmids and expression plasmids were performed as we have previously described [22], [24]. Briefly, TC620 cells were transfected with reporter constructs alone (200 ng) or in combination with expression plasmid(s) at 48 h prior to harvesting. When p65 siRNA was used, 50 pmol of Smartpool p65 siRNA (Dharmacon, Lafayette, CO) was included in the transfection as we have previously described [22]. Treatment with sodium butyrate (SB: 2 mM and 4 mM) was performed for 24 h prior to harvesting. Assay for luciferase was as previously described [24], [25].

### JCV infection assay

SVG-A is a human fetal glial cell line transformed by an origin-defective SV40 that expresses large T-antigen and supports JCV replication. SVG-A cells were transfected/infected with pJCV linearized by *Bam*HI digestion and harvested after 7 days for Western blot for viral VP1 and agnoprotein. In experiments to investigate the effect of dominant negative IκB, pCMV-IκBαM was included in the transfection with the total amount of DNA kept constant between samples. In experiments to investigate the effect of Rad51 inhibition, 15 µM RI-1 [34] was added following transfection.

### Immunocytochemistry (ICC)

TC620 cells were serum-starved overnight with 0.5% BSA and then either untreated or treated with 10 ng/ml TNF-α for 20 min. Cells were fixed in 4% paraformaldehyde in PBS for 10 min, washed, permeabilized for 5 min with 0.1% TritonX-100, blocked for 30 min with 5% normal goat serum and incubated 3 h at 37°C with rabbit anti-NF-κB p65 and mouse anti-Rad51 at a 1∶100 dilution in PBS. Cells were then washed, incubated for 2 h with secondary FITC-conjugated goat anti-rabbit and rhodamine-conjugated anti-mouse secondary antibodies at a 1∶200 dilution, washed, mounted with DAPI-containing mounting medium (VECTASHIELD, Vector Laboratories Inc. Burlingame, CA) and viewed by fluorescence microscopy.

### Cell fractionation

TC620 were transfected with pCMV-p65 and pCMV-Rad51 and the following day serum-starved overnight with 0.5% BSA and then either untreated or treated with 10 ng/ml TNF-α for 20 min. Nuclear and cytoplasmic fractions were prepared using the NE-PER nuclear and cytoplasmic reagents according to the Manufacturers protocol (Pierce Biotechnology, Rockford, IL) as we have previously described [33].

### ChIP assay

TC620 cells were transfected with JCV_E_-LUC, which contains the Mad-1 JCV NCCR in the presence or absence of expression plasmid for DN-IκB. The medium was changed to DMEM with 0.5% BSA and no serum. After overnight serum starvation, cells were stimulated with 10 ng/ml TNF-α for 20 min and ChIP performed at 48 h after transfection as we have previously described [22] using the ChIP assay kit (Upstate Cell Signaling Solutions). Cross-linking was performed with formaldehyde and the DNA sheared by sonication. The cells were lysed and immunoprecipitation was performed with antibody to Rad51, nonimmune rabbit serum or beads alone as indicated. DNA was extracted and PCR performed using primers spanning the JCV NCCR.

## Results

### Rad51 and NF-κB p65 cooperate to stimulate JCV early transcription

**Figure 1:**
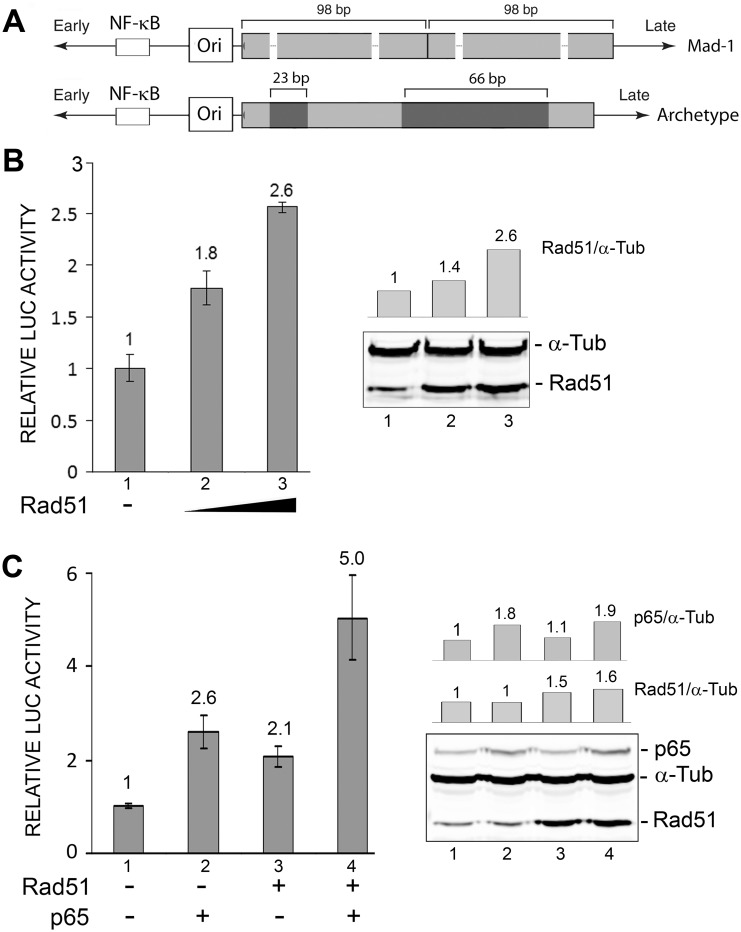
Effect of ectopic expression of Rad51 and NF-κB p65 on JCV early promoter reporter expression. **A.** Schematic representation (not to scale) of the Mad-1 neurovirulent form of JCV that was isolated from the brain of a PML patient and the archetypal form of JCV, which is found in the environment and may be the transmissible form of the virus. Relative to the archetype, Mad-1 contains 23 bp and 66 bp deletions (black boxes) in the late proximal region followed by a duplication of the remaining 98 bp sequence. Note that the NF-κB site lies in the highly conserved early proximal region of the JCV NCCR that lies between the early coding region (far left) and the viral origin of DNA replication (Ori). This region is highly conserved, not involved in rearrangements and present in all known strains of JCV. **B.** TC620 cells were transfected with luciferase reporter plasmid for the early promoter, JCV_E_-LUC in the presence or absence of increasing amounts of Rad51 expression plasmid (0, 0.5 and 1 µg). After 48 h, cells were harvested and assayed for luciferase activity. Activities were normalized to the activity for reporter alone (lane 1). The error bars represent one standard deviation. Expression of Rad51 was verified by Western blot as shown on the right-hand side of the panel with α-tubulin (α-Tub) as a loading control. Note the lane numbering in the Western blot corresponds to the numbering in the luciferase assay histogram on the left-hand side of the panel. The intensity of the Rad51 and α-Tub bands were measured for each lane and the ratio shown as a histogram above the Western. **C.** TC620 cells were transfected JCV_E_-LUC (left) in the presence or absence of Rad1 (0.5 µg) and/or NF-κB p65 (0.5 µg) expression plasmids. Expression of Rad51 and p65 were confirmed by Western blot with α-tubulin as a loading control, and this is shown on the right-hand side of the panel. Again, the lane numbering in the Western blot corresponds to the numbering in the luciferase assay histogram on the left-hand side of the panel. The intensity of the Rad51 and α-Tub bands were measured for each lane and the ratio shown as a histogram above the Western. The intensity of the Rad51, p65 and α-Tub bands were measured for each lane and the ratio of p65/α-Tub and Rad51/α-Tub are shown as histograms above the Western.

The JCV noncoding control region (NCCR) contains a binding site for NF-κB that is conserved in all strains of JCV (Fig. 1A) including the archetype and the rearranged neurovirulent strains of JCV that cause PML including the prototypical Mad-1 strain, which was used in this study. This unique NF-κB site mediates the stimulation of transcription caused by NF-κB p65 expression and treatment of cells with PMA or TNF-α [21]–[24]. This site is also regulated by the calcineurin/NFAT4 signaling pathway [25] and histone acetylation [26]. We now examine a role for the DNA repair protein Rad51 at this site. As shown in Fig. 1B, transient transfection of increasing amounts of Rad51 expression plasmid proportionately stimulated JCV early transcription measured using a luciferase reporter plasmid at 0.5 (p<0.05) and 1 µg (p<0.05). No effect was observed in the case of JCV late transcription (data not shown). An increasing level of Rad51 was verified by Western blot. As we have reported before [22], expression of NF-κB p65 also stimulated JCV early transcription (Fig. 1C, lane 2, p<0.05). When p65 was expressed with Rad51, there was an additive enhancement of JCV early transcription (Fig. 1C, lane 4, p<0.05). Each experiment was performed twice.

### Early promoter mutants defective in NF-κB binding fail to respond to Rad51

**Figure 2:**
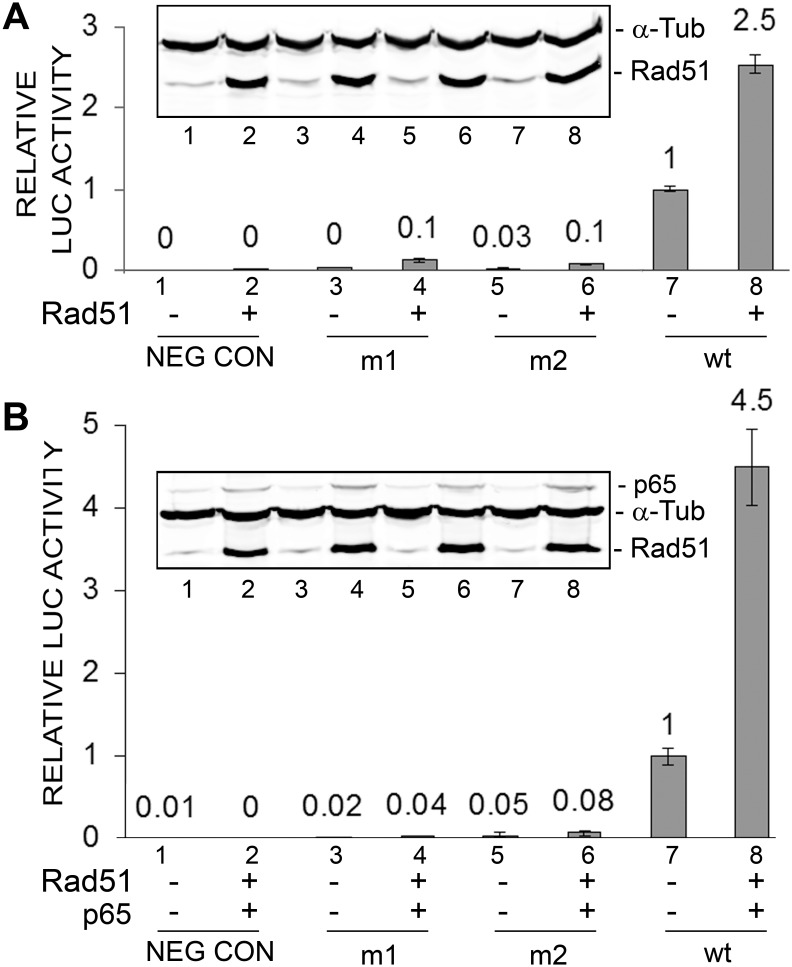
Effect of ectopic expression of Rad51 and p65 on JCV early promoter mutant reporters expression. **A.** TC620 cells were transfected with JCV_E_-LUC reporter plasmid containing either the m1 or m2 NF-κB site mutation or with wild-type (wt) promoter in the presence or absence of Rad51 (1 µg). After 48 h, cells were harvested and assayed for luciferase activity. Activities were normalized to the activity for wild-type alone (lane 7). The error bars represent standard deviation. Western blot is shown as an inset subpanel within the luciferase activity histogram. The lane numbering in the Western blot corresponds to the numbering in the luciferase assay histogram. The intensity of the Rad51 and α-Tub bands were measured for each lane and the ratio shown as a histogram above the Western. **B.** TC620 cells were transfected as in Panel A except that expression plasmids for Rad51 (0.5 µg) and p65 (0.5 µg) were used together.

In earlier studies, we produced two mutant early promoters (m1 and m2) defective at the NF-κB p65-binding site [22]. The two mutants had lower rates of basal transcription and response to Rad51 (Fig. 2A, lanes 3–6) while the wild-type promoter had a higher basal rate of transcription (lane 7) and was robustly inducible by Rad51 (lane 8). The activity of the basal wild-type JCV early LUC reporter promoter was significantly higher (about four-fold) than either m1 or m2 (Fig. 2A, lane 7) as was the activity of wild-type in the presence of Rad51 (Fig. 2A, lane 8). Similarly, the same pattern of response was seen when Rad51 was added together with p65 (Fig. 2B). This experiment was performed twice.

### Small interfering RNA to NF-κB p65 inhibits Rad51-stimulated and Rad51/NF-κB p65-stimulated transcription of the JCV early promoter

**Figure 3:**
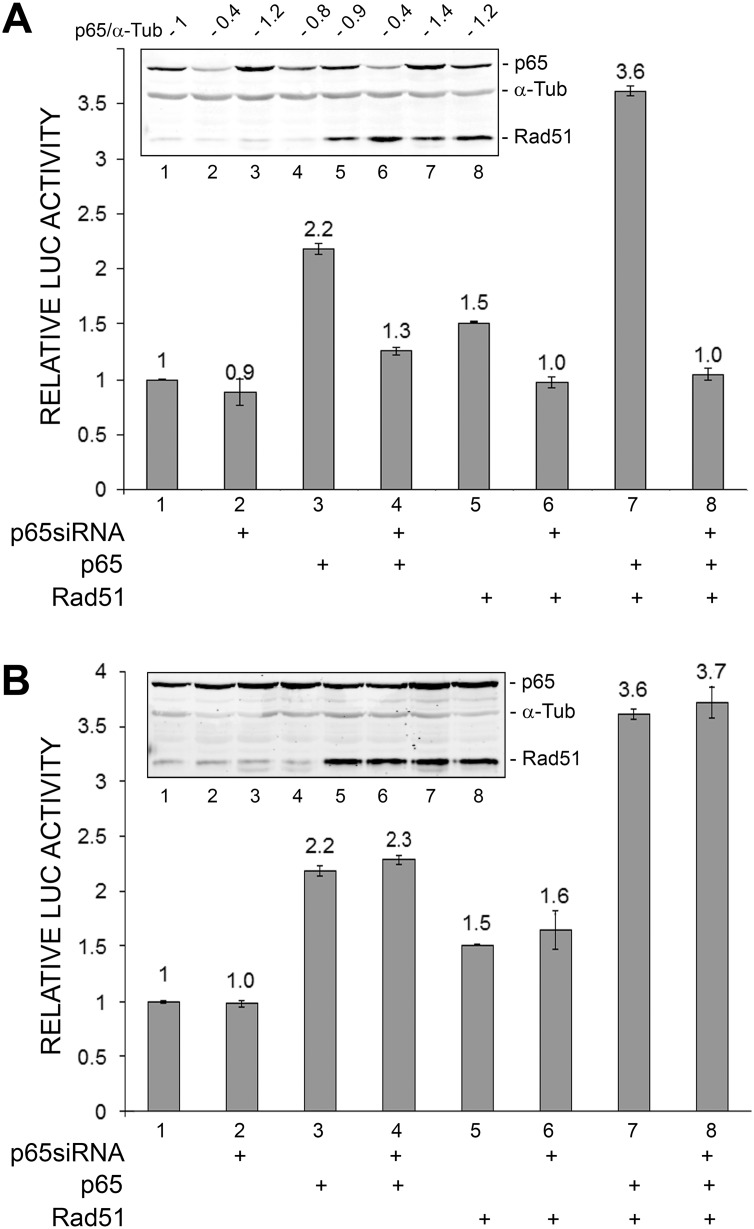
Effect of ectopic expression of Rad51 and siRNAs on JCV early promoter reporter expression. **A.** TC620 cells were transfected with JCV_E_-LUC reporter plasmids and expression plasmids for Rad51 (0.5 µg) and p65 (0.5 µg) and/or siRNA for p65 (200 nmol) in various combinations as indicated. After 48 h, cells were harvested and assayed for luciferase activity. Activities were normalized to the activity for reporter alone. The error bars represent standard deviation. Western blot is shown as an inset subpanel within the luciferase activity histogram. The lane numbering in the Western blot corresponds to the numbering in the luciferase assay histogram. The ratio of the quantified relative intensities of the p65 to the α-tubulin bands for each lane are given above the blot to indicate the extent of p65 knockdown. **B.** As for Panel A except that non-targeting (NT) siRNA (200 nmol) replaced p65 siRNA. This was performed in the same experiment as Panel A but is shown separately for clarity of presentation.

To confirm a role for NF-κB in the stimulation of JCV early transcription, we employed siRNA to p65 (Fig. 3). Stimulation of JCV early transcription by p65 or Rad51 or both was inhibited (Fig. 3A) but a non-targeting siRNA used in the same experiment was without effect (Fig. 3B). This experiment was performed twice.

### A dominant negative mutant of IκBα inhibits p65 and Rad51 stimulation of JCV_E_ transcription

**Figure 4:**
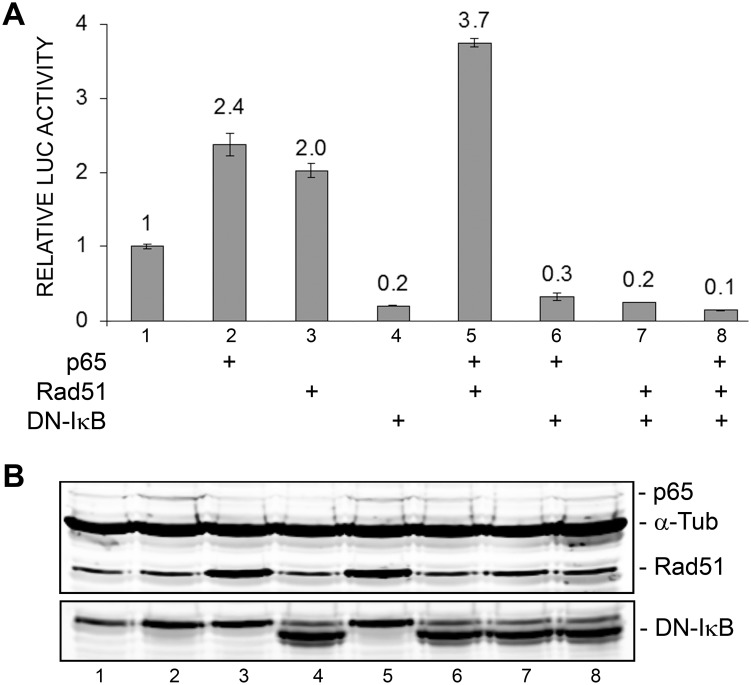
Effect of ectopic expression of dominant negative ΔN-IκB on Rad51 and p65 stimulation of JCV early promoter reporter expression. **A.** TC620 cells were transfected with JCV_E_-LUC in the presence or absence of Rad51 (0.5 µg), p65 (0.5 µg) and/or DN-IκB (0.5 µg) expression plasmid in various combinations as indicated. After 48 h, cells were harvested and assayed for luciferase activity. Activities were normalized to the activity for reporter alone. The error bars represent standard deviation. **B.** Expression of Rad51, p65 and ΔN-IκB in this experiment was confirmed by Western blot with α-tubulin as a loading control (lower panel). The lane numbering in the Western blot corresponds to the numbering in the luciferase assay histogram.

IκBα is a cytoplasmic proteins that binds and sequesters inactive NF-κB. Phosphorylation of IκBα by an upstream kinase renders it susceptible to ubiqutination and subsequent degradation by the proteasome, which thus frees active NF-κB to translocate to the nucleus. In the dominant negative IκB mutant, DN-IκB, substitution for alanine of the two serine residues that are phosphorylated by upstream kinase render it resistant to phosphorylation, ubiqutination and degradation by the proteasome thus preventing activation NF-κB [35]. As expected, DN-IκB inhibited p65-stimulated transcription (Fig. 4A, compare lanes 2 and 6). It also inhibited Rad51-stimulated transcription (lanes 3 and 7) and transcription stimulated by both (lanes 5 and 8). Expression of p65, Rad51 and DN-IκB was confirmed by Western blot (Fig. 4B). These data support a role for NF-κB p65 in mediating the stimulation of JCV early transcription by Rad51. This experiment was performed twice.

### C/EBPβ LIP isoform inhibits basal, Rad51-stimulated and Rad51/NF-κB p65-stimulated transcription of the JCV early promoter

**Figure 5:**
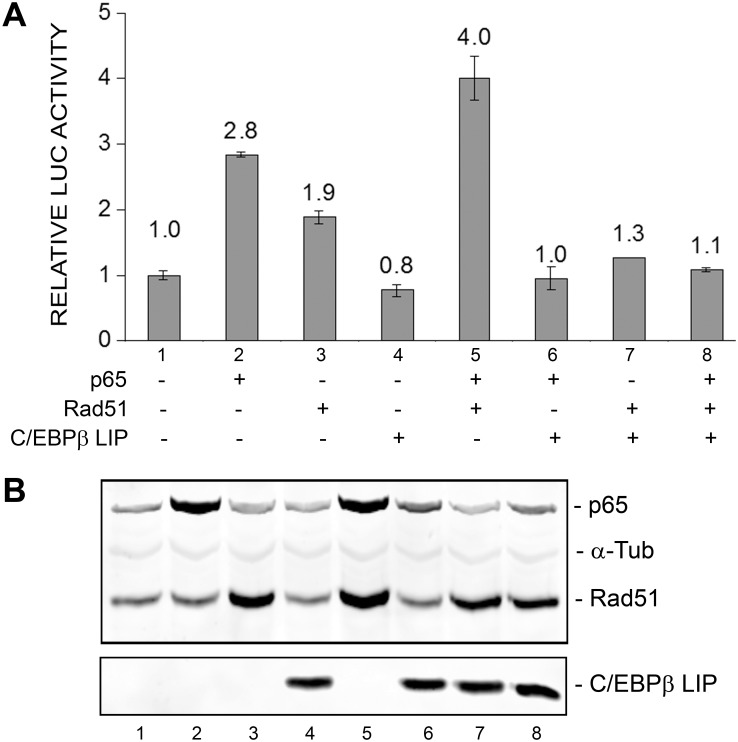
Effect of ectopic expression of Rad51 and C/EBPβ on JCV early promoter reporter expression. **A.** TC620 cells were transfected with JCV_E_-LUC in the presence or absence of Rad51 (0.5 µg), p65 (0.5 µg) and/or C/EBPβ LIP (0.5 µg) expression plasmid in various combinations as indicated. After 48 h, cells were harvested and assayed for luciferase activity. Activities were normalized to the activity for reporter alone. The error bars represent standard deviation. **B.** Expression of Rad51, p65 and LIP were confirmed by Western blot with α-tubulin as a loading control (lower panel). The lane numbering in the Western blot corresponds to the numbering in the luciferase assay histogram.

In addition to the positive effect of p65 at the NF-κB site, we have reported that the transcription factor C/EBPβ, especially the LIP isoform, binds near to the same site and inhibits JCV transcription [22]. Thus it was of interest to investigate the effects of C/EBPβ LIP and Rad51, each alone and in combination on JCV early transcription. We found that C/EBPβ LIP inhibited both Rad51- and p65-stimulated of JCV early promoter (Fig. 5). This experiment was performed twice.

### Rad51 and sodium butyrate cooperate to stimulate JCV early transcription

**Figure 6:**
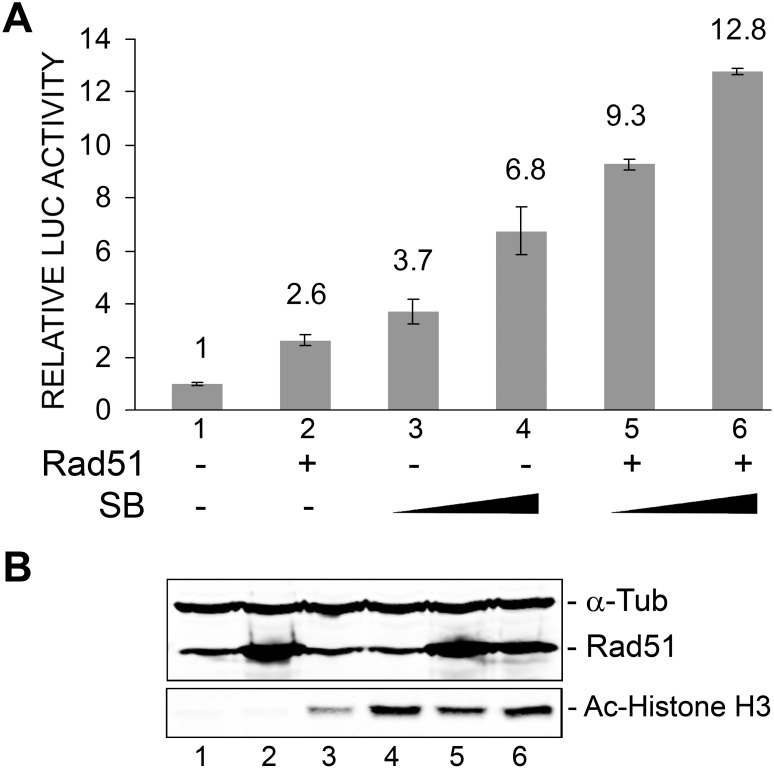
Effect of ectopic expression of Rad51 and sodium butyrate treatment on JCV early promoter reporter expression. TC620 cells were transfected with JCV_E_-LUC in the presence or absence of Rad51 expression plasmid (1 µg), and/or treated with SB (2 mM and 4 mM) as indicated. **A.** After 48 h, cells were harvested and assayed for luciferase activity. Activities were normalized to the activity for reporter alone. The error bars represent standard deviation. **B.** The Western blots for Rad51, acetyl-histone H3 (K9) and α-tubulin are shown. The lane numbering in the Western blot corresponds to the numbering in the luciferase assay histogram in Panel A.

Since we recently found that the activity of the JCV early promoter is stimulated by histone deacetylase inhibitors such as sodium butyrate (SB) and trichostatin A and this effect is mediated through the NF-κB site [26], it seemed possible that Rad51 and sodium butyrate might act together to enhance JCV early transcription since they act at the same site. We next examined the effect of SB treatment on the Rad51 stimulation of the early promoter. The effect of SB on transcription was markedly more pronounced in the presence of Rad51 (Fig. 6A). We conclude that Rad51 and sodium butyrate, which both act at the NF-κB site of the JCV NCCR, cooperate to stimulate JCV early transcription. The level of Rad51 was measured by Western blot and was confirmed to increase upon transfection of expression plasmid (Fig. 6B, lanes 2, 5 and 6). Protein acetylation was assessed by Western blot for acetylated Histone H3 (K9) and was markedly increased upon sodium butyrate treatment (Fig. 6B, lanes 3–6) as expected. This experiment was performed twice.

### Inhibition of NF-κB by IκBDN expression or Rad51 by RI-1 treatment inhibits JCV infection

**Figure 7:**
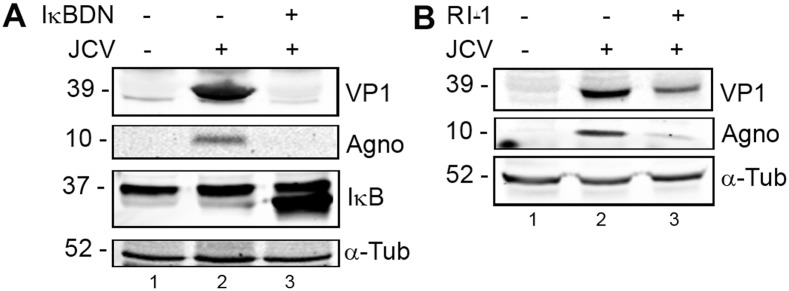
Effect of ectopic expression of IκBDN and RI-1 on JCV infection. A. SVGA cells (SV40 T-Ag-transformed human fetal glial cells) were transfected/infected with JCV with and without expression plasmid for IκBDN as indicated and harvested after 7 d for Western blot. **B.** SVGA cells were transfected/infected with JCV with and without 15 µM RI-1 Rad51 inhibitor and harvested after 7d for Western blot.

To investigate the role of NF-κB in JCV infection, we performed transfection/infection experiments with SVGA (an SV40 T-Ag-transformed human fetal glial cell line) and wild-type JCV Mad-1 genomic DNA in the presence and absence of expression plasmid for IκBDN (Fig. 7A). After 7 days, expression levels of VP1 and agnoprotein were measured by Western blot. The expression of both proteins was reduced in the presence of IκBDN showing that NF-κB is important in the JCV infection. To investigate the role of Rad51 in JCV infection, we performed transfection/infection experiments with SVGA with JCV and treated cells with and without RI-1. RI-1 covalently and irreversibly binds to Rad51 at Cys319, which is essential for filament formation and recombinase activity [34]. After 7 days, expression levels of VP1 and agnoprotein were measured by Western blot (Fig. 7B). The expression of both proteins was reduced in the presence of RI-1 showing that Rad51 is important in the JCV infection. Note here that Rad51 plays an essential role in cellular proliferation and Rad51 inhibitors are known to slow down cell growth. In the case of SVG-A cells, 15 µM RI-1 that was used in the experiment has no effect on cell viability but causes up to a 45–50% reduction in the rate of cell proliferation as measured by MTT assay depending on cell density (data not shown). However, we normalized all samples for total cell protein before gel loading to compensate for this. Hence, the level of the cellular structural protein α-tubulin is constant and differences in VP1 and agnoprotein are therefore due to specific effects of RI-1 on viral protein expression levels.

### Translocation of Rad51 to the nucleus after cytokine induction

**Figure 8:**
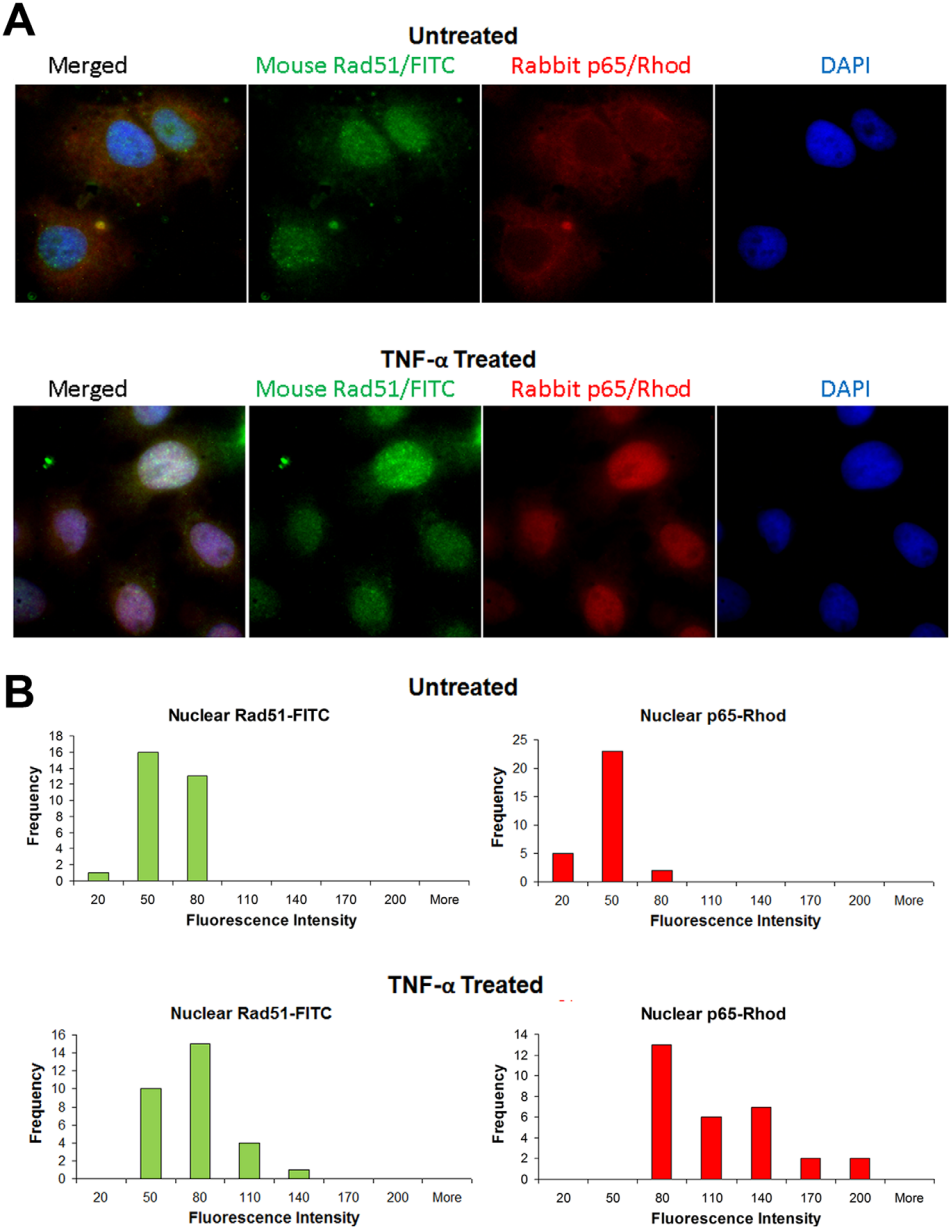
Effect of TNF-α on subcellular localization of NF-κB p65 and Rad51: immunocytochemistry. **A.** TC620 cells were either untreated or treated with 10 ng/ml TNF-α for 20 min and subject to ICC with antibodies to p65 and Rad51, washed, mounted with DAPI-containing mounting medium and viewed by fluorescence microscopy as described in Materials and Methods. Note, these cells were not transfected so the fluorescence is due to endogenous p65 and Rad51. **B.** Quantification of immunocytochemistry images: Images of labeled cells were analyzed using Adobe Photoshop CS. Nuclear areas of the labeled cells were manually outlined using the lasso selection tool based on DAPI labeling and the average fluorescence intensity levels for the red and green channels were recorded from 30 nuclei for each condition. Next, the fluorescence intensity levels data were converted into histograms using the Analysis Toolpack add-in of Microsoft Excel.

**Figure 9:**
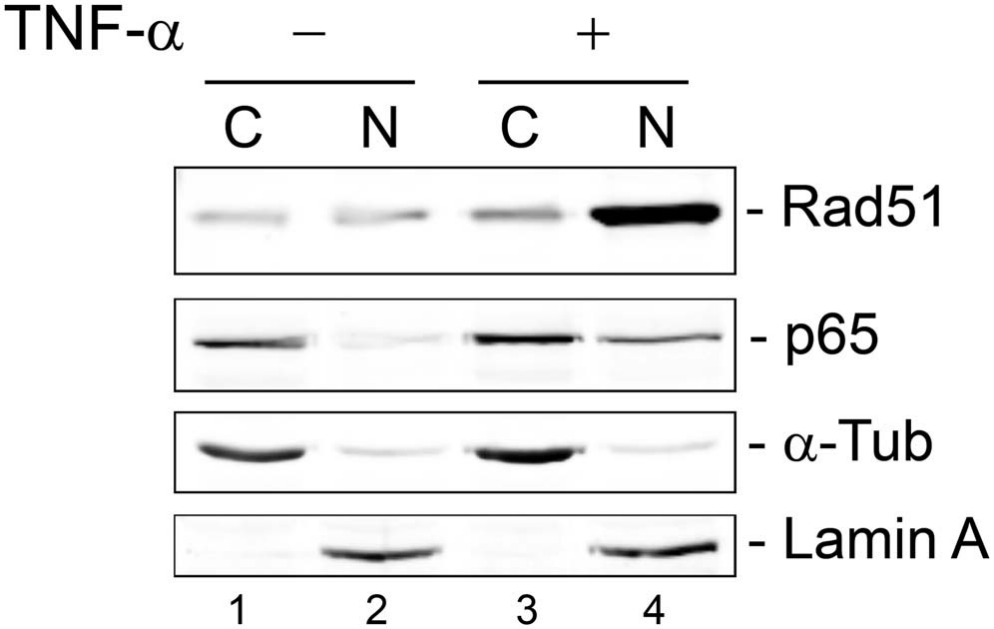
Effect of TNF-α on subcellular localization of NF-κB p65 and Rad51: Cell fractionation. TC620 cells were transfected with expression plasmids for p65 and Rad51, the next day serum-starved overnight and then either untreated or treated with 10 ng/ml TNF-α for 20 min and subject to cell fractionation as described in Materials and Methods.

Inactive NF-κB resides in the cytoplasm in association with the inhibitory protein I-κB and is released after cell stimulation by pro-inflammatory cytokines to migrate to the nucleus. Since we have found that Rad51 and NF-κB function together, we next performed immunocytochemistry (ICC) to investigate whether there was any correlation between changes in the localization of these two proteins under different conditions. TC620 cells were serum starved overnight, untreated or treated with 10 ng/ml TNF-α for 20 min and subject to ICC as described in Materials and Methods (Fig. 8). As expected, treatment with TNF-α resulted in translocation of p65 to the nucleus and this was also found to be the case for Rad51. Thus changes in Rad51 localization correlate with those of NF-κB suggesting the involvement of a common process and this may be important for the costimulation of JCV gene expression by NF-κB and Rad51. Interestingly, the nuclear Rad51 labeling showed a slight degree of speckling reminiscent of Rad51 nuclear foci formation although no DNA damaging agents were used in this experiment. Similar results were obtained using cell fractionation of TC620 cells that had been transfected with Rad51 and p65 expression plasmids (Fig. 9). Surprisingly, the degree of redistribution of Rad51 to the nucleus in this experiment (compare lanes 2 and 4) was greater than that of NF-κB suggesting other additional mechanisms might exist for Rad51 translocation to the nucleus.

### Effect of TNF-α-stimulated binding of Rad51 to the JCV NCCR in vivo by DN-IκB

**Figure 10:**
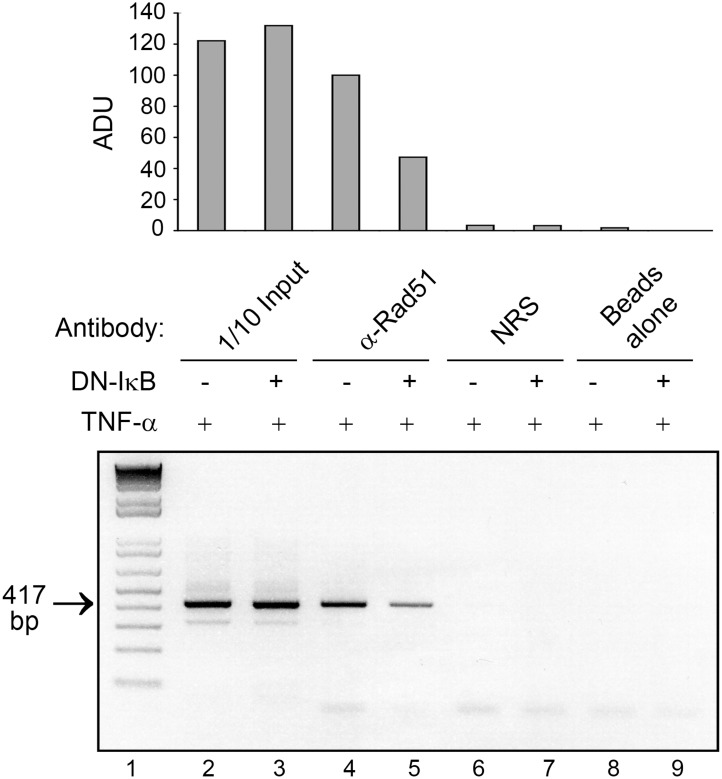
Effect of DN-IκB on TNF-α-stimulated binding of Rad51 to the JCV NCCR in vivo. TC620 cells were transfected with JCV_E_-LUC plasmid containing the JCV NCCR in the presence and absence of expression plasmid for DN-IκB, starved of serum and stimulated with TNF-α as described in Materials and Methods. Following cross-linking, ChIP assay was performed using primers flanking the JCV NCCR and antibody to Rad51, normal rabbit serum or no antibody (beads alone). The position of the 417 base pair band corresponding to the amplified NCCR is indicated by an arrow. Lane 1 - molecular weight markers. The top panel shows quantification of the intensity of each band expressed as absorption density units (ADU) after normalizing the density for Rad51 in the absence of DN-IκB (lane 4) to 100%.

The translocation of p65 and Rad51 in response to TNF-α suggested that Rad51 might associate with the JCV NCCR after activation of the NF-κB pathway. We stimulated cells with TNF-α in the presence and absence of DN-IκB, which is a dominant negative inhibitor of NF-κB signaling. As shown in Figure 10, Rad51 binds to the JCV NCCR in ChIP assay and this binding is reduced about 2-fold by DN-IκB (compare lane 5 to lane 4). These data indicate that Rad51 that is translocated into the nucleus in response to NF-κB signaling interacts with the JCV NCCR in vivo.

## Discussion

Our data indicate that the double-strand DNA break repair protein Rad51 stimulates transcription of the JCV early promoter and evidence is presented for the involvement of the transcription of factor NF-κB in this process. NF-κB is a key regulatory protein in the life cycle of JCV and controls the activation of JCV transcription by cytokines such a TNF-α [24], co-ordinates signaling pathways that converge at the NF-κB-binding site such as calcineurin/NFAT4 [25] and mediates epigenetic control of the virus by protein acetylation/deacetylation [26]. In the present study, the stimulation of JCV early transcription by Rad51 was enhanced when it was co-expressed in the presence of NF-κB p65 and ablated by mutation in the JCV NF-κB binding site. Rad51 stimulation of JCV early transcription was also inhibited by transfection of siRNA to p65 or expression of a dominant negative form of IκBα, which sequesters NF-κB in an inactive state in the cytoplasm. Taken together, these data implicate NF-κB as the mediator of the effects of Rad51 on JCV early transcription.

Interestingly, we have also found that Rad51 has a similar role on the HIV-1 virus and acts to stimulate HIV-1 transcription by binding to NF-κB p65 and activating the NF-κB site in the HIV-1 LTR [29], [30]. In these studies, we found that there was association of Rad51 with NF-κB as detected by immunoprecipitation/Western blot with cell extracts expressing endogenous levels of both proteins. Results from GST pull-down protein-binding assays showed that p65/NF-κB interacts with Rad51 protein while analysis of a series of GST-Rad51 deletion mutants demonstrated the importance of the region of Rad51 between amino acids 40–80 in this interaction [29].

What is the importance of this interaction in the regulation of the JCV life cycle? In earlier studies, we found that infection of glial cells by JCV causes extensive DNA damage and elicits the cellular DNA damage response (DDR). JCV-infected cells exhibit increased ploidy in metaphase spreads correlating with duration of infection as well as increased micronuclei formation and the presence of γH2AX, which are indicative of DNA damage [28]. Western blot analysis revealed that JCV infection perturbed the expression of some DNA repair proteins including a large elevation in the level of Rad51 [28]. This induction of Rad51 was detected in Western blot of JCV-infected primary glial cell cultures and also in immunohistochemistry of PML clinical samples. Since Rad51 stimulates JCV gene expression, the induction of Rad51 expression by DDR following JCV infection may be a positive feedback mechanism whereby viral activity is boosted after infection.

We have been investigating the role of NF-κB in JCV reactivation and provided evidence to support our hypothesis that extracellular cytokines that initiate signal transduction through pathways that activate NF-κB, which turns on JCV gene expression. Normally, NF-κB is sequestered in an inactive form in the cytoplasm by an inhibitory molecule, IκB, but is released upon cellular stimulation and enters the nucleus [36]. In the case of JCV, active NF-κB strongly stimulates both early and late transcription and serves as a control nexus for other signaling pathways to modulate viral activity in response to cytokine stimulation [24], interaction with other transcription factors including C/EBPβ [22] and NFAT4 [25], and epigenetic modulation through protein acetylation [26]. Interestingly, the DNA damage response (DDR) is known to result in the activation of the NF-κB signaling pathway, a phenomenon known as nucleus to cytoplasm or “inside-out” NF-κB signaling [37], [38]. Thus, JCV infection can result in both the induction of Rad51 expression and the activation of the NF-κB pathway. Since Rad51 and activated NF-κB act together to stimulate JCV gene expression, this might provide a powerful positive feedback loop in the reactivation of the virus.

In conclusion, our evidence suggests that NF-κB is a crucial control nexus where different signals can converge and may represent a switch for the initiation of viral reactivation. Recently, experimental and mathematical modeling of the NF-κB signaling module have provided evidence for the existence of a threshold level of input giving a switch-like response [39] and it is possible that such a mechanism triggers the switch between silent JCV and JCV reactivation. This would be an interesting area for future research to advance our understanding of the JCV life cycle, PML pathogenesis and possible novel targets for therapeutic intervention.
